# Comprehensive Analysis of Necroptosis-Related Genes as Prognostic Factors and Immunological Biomarkers in Breast Cancer

**DOI:** 10.3390/jpm13010044

**Published:** 2022-12-26

**Authors:** Yingkun Xu, Qiulin Wu, Zhenrong Tang, Zhaofu Tan, Dongyao Pu, Wenhao Tan, Wenjie Zhang, Shengchun Liu

**Affiliations:** Department of Breast and Thyroid Surgery, The First Affiliated Hospital of Chongqing Medical University, Chongqing 400042, China

**Keywords:** TCGA, breast cancer, necroptosis, risk model, GSEA

## Abstract

Breast cancer (BC) is a lethal malignancy with a poor prognosis. Necroptosis is critical in the progression of cancer. However, the expression of genes involved in necroptosis in BC and their association with prognosis remain unclear. We investigated the predictive potential of necroptosis-related genes in BC samples from the TCGA dataset. We used LASSO regression to build a risk model consisting of twelve necroptosis-related genes in BC. Using the necroptosis-related risk model, we were able to successfully classify BC patients into high- and low-risk groups with significant prognostic differences (*p* = 4.872 × 10 ^−7^). Additionally, we developed a matched nomogram predicting 5, 7, and 10-year overall survival in BC patients based on this necroptosis-related risk model. Our next step was to perform multiple GSEA analyses to explore the biological pathways through which these necroptosis-related risk genes influence cancer progression. For these twelve risk model genes, we analyzed CNV, SNV, OS, methylation, immune cell infiltration, and drug sensitivity in pan-cancer. In addition, immunohistochemical data from the THPA database were used to validate the protein expression of these risk model genes in BC. Taken together, we believe that necroptosis-related genes are considered potential therapeutic targets in BC and should be further investigated.

## 1. Introduction

Globally, breast cancer is one of the most common malignancies diagnosed in women, a heterogeneous disease comprising multiple subtypes that exhibit distinct histopathological features, molecular alterations, and clinical course [1,2]. Although advanced treatments have significantly improved clinical outcomes, recurrence and metastasis remain the leading causes of breast cancer. The prognosis of breast cancer patients is poor due to the lack of effective therapeutic targets. Therefore, further understanding of the molecular mechanisms is urgently needed to identify molecular targets for breast cancer therapy [3].

As early as the 19th century, medical researchers began to pay attention to the morphological characteristics of cell death, and proposed that there are only two modes of cell death, apoptosis and necrosis. They believed that apoptosis was a regulated cell death method, and that necrosis was an unregulated way of cell death [4]. Apoptosis is crucial in promoting embryonic development, maintaining body homeostasis, developing immune cells, and removing uncontrolled cells to avoid tumorigenesis [5]. Apoptosis is tightly regulated by intracellular signal transduction factors so that it can respond to the stimulation of specific physiological and pathological characteristics in a regulated manner. When apoptosis occurs, the apoptotic cells form apoptotic bodies, the cell membrane remains intact, and the cytoplasmic contents are not released outside the cell membrane, and thus do not trigger an inflammatory response.

In contrast, researchers have long believed that cell necrosis is passive death due to specific pathological conditions, such as chemical or physical damage, lack of nutrients, and cellular hypoxia. The cell membrane permeability of necrotic cells increases, resulting in cell swelling, organelle deformation or enlargement, and, finally, cell rupture, which causes the release of cell contents and further induces an inflammatory response [6]. Researchers delved into cell death and realized that not all necrosis is passive [7]. Cell death due to pathophysiological stimuli can also display morphological features of necrosis under certain conditions. In 2005, Alexei Degterev et al. discovered a cell death mode which Necrostain-1 could regulate, and named this new cell death mode necroptosis [8]. It is important to know that receptor-binding serine/threonine-protein kinase 3 (RIPK3) and its substrate, mixed lineage kinase domain-like protein (MLKL), play an integral role in necroptosis. Translating transformed MLKL to the inner side of the cell membrane and disrupting the integrity of the cell membrane is an essential step for necroptosis [9].

Previous studies have found that necroptosis is prevalent in mouse breast cancer necrotic areas, and the phosphorylation level of mixed lineage kinase domain-like protein (MLKL) is significantly increased in late breast cancer. It has been suggested that MLKL-mediated necroptosis in tumor cells promotes lung metastasis of breast cancer. Since necroptosis is pro-inflammatory cell death, the production of inflammatory cytokines was significantly reduced in macrophages of MLKL-deficient tumors, suggesting that the inflammatory response induced by necroptosis may contribute to breast cancer lung metastasis [10,11]. In addition, another study found that the knockdown of RIPK3 expression in recurrent breast cancer cells inhibited the proliferation of breast cancer cells and suppressed the activity of YAP/TAZ [12].

**Figure 1:**
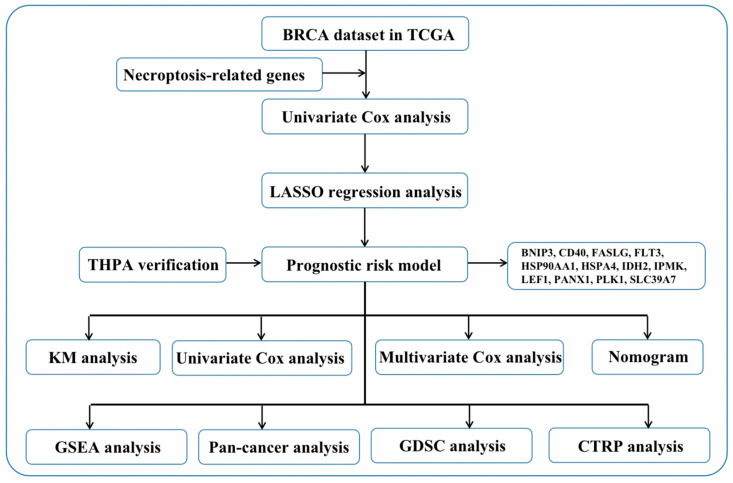
The flow chart of this research.

This study comprehensively used multiple bioinformatic analyses to characterize the potential biological roles of aberrant necroptosis-related genes in BC progression, and successfully constructed a new prognostic risk model in BC. This prognostic risk model consists of twelve necroptosis-related genes, BNIP3, CD40, FASLG, FLT3, HSP90AA1, HSPA4, IDH2, IPMK, LEF1, PANX1, PLK1, and SLC39A7. In addition, to help readers understand the idea of this research more clearly, we created a flow chart for this research (Figure 1). Furthermore, we assessed this prognostic risk model’s biological role and clinical relevance in because, and provided information on patient prognosis, immunohistochemistry, immune cell infiltration, and drug sensitivity.

## 2. Materials and Methods

### 2.1. Acquisition of Necroptosis-Related Data and Gene Selection

In January 2022, we downloaded breast cancer mRNA-seq (FPKM) data from the official website of the TCGA database (http://portal.gdc.cancer.gov/ (accessed on 1 January 2022)). The samples included 1109 breast cancer tissues and 113 normal tissues. For further analysis, gene expression data were normalized and log2 was transformed. The clinicopathological feature information of breast cancer patients was downloaded and extracted from the TCGA database, including age, gender, clinical stage, and survival time, and samples with missing clinical information were deleted. The necroptosis-related genes were derived from the GSEA database, as previous literature has reported. Supplementary Table S1 provides detailed statistics on 67 necroptosis-related genes used in this study.

### 2.2. Processing and Analyzing Data

To generate a heatmap of necroptosis-related gene expression, we used RNA-seq transcriptomic data from the BC dataset in the TCGA database, as well as the “pheatmap” extension package. In addition, the “limma” extension package can be used to analyze necroptosis differential expression of apoptosis-related genes. We then performed a univariate Cox regression curve analysis of these necroptosis-related molecules in BC to determine their prognostic value. To determine LASSO regression curves, we used the R language extensions “glmnet” and “survival” to perform regression curve analysis, and to draw survival curves. ROC curve analysis was performed using the “survivalROC” extension package to verify the predictive accuracy of the risk model. In addition to the clinicopathological data, the correlation between the risk model and the pathological characteristics of BC patients was also analyzed. With the “survival” and “forestplot” extensions, we performed univariate and multivariate Cox regression curve analysis. After synthesizing various risk factors, we used the “rms” extension package to construct the corresponding nomograms to facilitate future clinical diagnoses and treatments. We also carried out multiple GSEA analyses using the “plyr”, “ggplot2”, “grid”, and “gridExtra” extensions to explore the biological pathways affected by risk model genes in BC.

### 2.3. Network Construction and Analysis of Protein Interactions

The STRING database (http://string-db.org/ (accessed on 1 January 2022)) searches for known protein–protein and predicted protein–protein interactions, and necroptosis-related genes were analyzed in the STRING online database for protein–protein interactions [13,14]. The protein–protein interaction (PPI) network analysis was performed, and then the information of the PPI network was imported into Cytoscape 3.9.0 (https://cytoscape.org/ (accessed on 1 January 2022)) for visualization. As one of the open-source software tools for bioinformatics analysis, Cytoscape is used to visualize and explore biological networks composed of proteins, genes, and other types of interactions, and is one of the essential tools for bioinformatics research [15].

### 2.4. GSCA Database

The GSCA database, developed by the research group of Professor Guo Anyuan from Huazhong University of Science and Technology, provides a series of online tools to perform genomics and immunogenomic analysis [16]. Based on this database, we can combine clinical information and small molecule drugs to mine candidate biomarkers and valuable small drugs for better experimental design and further clinical trials. In this study, we performed OS, CNV, SNV, methylation, and immune cell infiltration analysis of risk model genes BNIP3, CD40, FASLG, FLT3, HSP90AA1, HSPA4, IDH2, IPMK, LEF1, PANX1, PLK1, and SLC39A7 using the online analysis tool of the GSCA database. We also used the GDSC and CTRP databases to explore the drug sensitivity of these necroptosis-related genes (http://bioinfo.life.hust.edu.cn/GSCA/#/ (accessed on 1 January 2022)).

### 2.5. The Human Protein Atlas Database

In the THPA database, transcriptomic and proteomic techniques are used to study protein expression in human tissues and organs based on RNA and protein levels (https://www.proteinatlas.org/ (accessed on 1 January 2022). Immunohistochemistry was used to examine the distribution and expression of each protein in 48 normal human tissues, 20 tumor tissues, 47 cell lines, and 12 blood cells [17,18]. THPA database was used to investigate the differential expression of necroptosis-related genes in normal and cancerous breast tissue.

### 2.6. Statistical Analysis

R 4.1.1 and Strawberry Perl were used for the statistical analysis. The expression of necroptosis-related genes in breast cancer versus normal breast tissue was analyzed using the Wilcox non-parametric rank test (Wilcox Test). We analyzed the relationship between gene expression and clinicopathological characteristics using the χ^2^ test. The Kaplan–Meier method was used to analyze the relationship between gene expression and survival of breast cancer patients, while univariate and multivariate Cox regression analysis was used to analyze the significance of the risk model in breast cancer patients. *p* < 0.05 indicated statistical significance.

## 3. Results

### 3.1. The Expression and Interaction of Necroptosis-Related Molecules in BC

**Figure 2:**
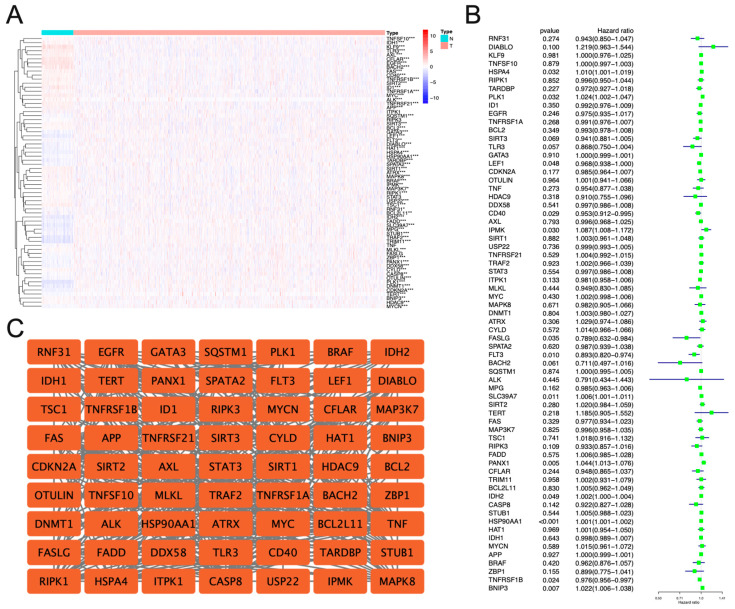
Expression and interaction of necroptosis-related molecules in BC. (**A**) Heat map showing the expression of target necroptosis-related genes in breast cancer. * *p* < 0.05, ** *p* < 0.01, and *** *p* < 0.001. (**B**) The forest plot shows the univariate Cox analysis of necroptosis-related genes in breast cancer. (**C**) This protein–protein interaction network shows how target necroptosis-related genes interact.

During carcinogenesis, cancer researchers’ abnormally expressed genes tend to receive greater attention than the genes that do not change significantly. To observe whether the expression of these 67 necroptosis-related genes is different in breast cancer, we extracted the mRNA expression profiles of these 67 necroptosis-related genes from the TCGA database. We drew a heat map to make it more intuitive to demonstrate differences in mRNA expression (Figure 2A). Except for the five genes ITPK1, RIPK3, STAT3, TNF, and FASLG, the remaining 62 genes showed significant differences in expression between breast cancer and normal breast samples. The forest plot drawn by the subsequent univariate Cox regression analysis showed that genes such as HSP90AA1, PANX1, BNIP3, FLT3, SLC39A7, TNFRSF1B, CD40, IPMK, PLK1, HSPA4, FASLG, LEF1, and IDH2 play a critical role in the progression of breast cancer (Figure 2B). Since protein molecules encoded by genes carry out most biological processes in organisms, we drew a network diagram showing the interactions between proteins encoded by these necroptosis-related genes (Figure 2C).

### 3.2. Using Necroptosis-Related Genes to Construct Risk Models and Correlations of Clinicopathological Features in BC

**Figure 3:**
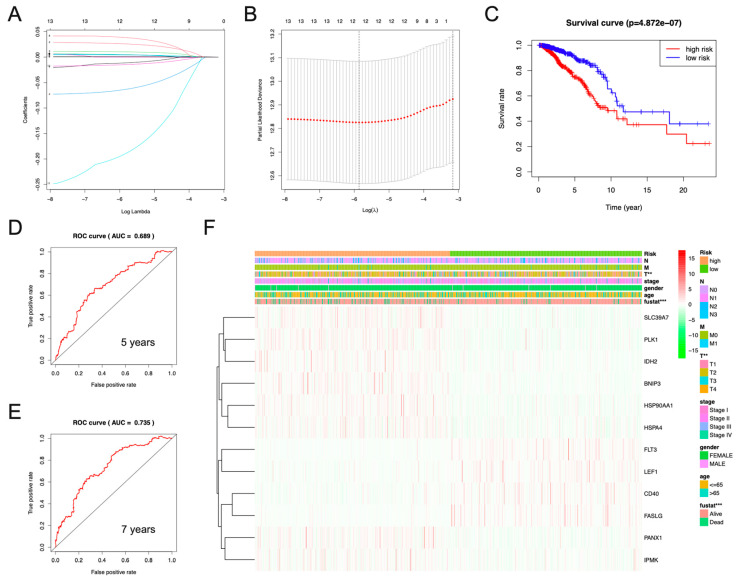
Using necroptosis-related genes to construct risk models and correlations of clinicopathological features in BC. (**A**,**B**) Lasso coefficients and vertical dashed lines were calculated at the best log (lambda) value, and coefficients of prognostic-related genes were displayed. (**C**) The K-M curve of breast cancer patients showed that the low-risk group had a significantly higher survival rate than the high-risk group based on this necroptosis-related risk model (*p* = 4.872 × 10 ^−7^). In particular, blue represents the low-risk group and red represents the high-risk group. (**D**,**E**) ROC curves were drawn based on this necroptosis-related risk model, with AUC values representing 5- and 7-year OS in breast cancer patients. (**F**) The heatmap illustrating the correlation between necroptosis-related risk models and clinicopathological characteristics of breast cancer patients. ** *p* < 0.01; *** *p* < 0.001.

By using LASSO regression curve analysis to build a model consisting of BNIP3, CD40, FASLG, FLT3, HSP90AA1, HSPA4, IDH2, IPMK, LEF1, PANX1, PLK1, and SLC39A7, we were able to fully exploit the prognostic value of these necroptosis-related genes in breast cancer (Figure 3A,B). Based on the algorithm of this model, the corresponding survival curve displayed shows that the survival rate of breast cancer patients in the high-risk group was significantly lower than that of breast cancer patients in the low-risk group (*p* = 4.872 × 10 ^−7^) (Figure 3C). In order to test the predictive accuracy of this necroptosis-related risk model, we performed ROC curve analysis, which showed that the five-year AUC was 0.689 and the seven-year AUC was 0.735 (Figure 3D,E). As a result, the necroptosis-related risk model seems to have a good predictive ability. Subsequently, we performed a correlation analysis between the necroptosis-related risk model and clinicopathological features, and the analysis results were presented in the form of a heat map. There was a significant correlation between this breast cancer risk model and T, fustat (Figure 3F). The formula for calculating the risk model is as follows:

Risk model = 0.01025 × BNIP3 + 0.0005137 × HSP90AA1 + 0.004524 × HSPA4 + 0.0007724 × IDH2 + 0.03849 × IPMK + 0.02598 × PANX1 + 0.004856 × PLK1 + 0.004228 × SLC39A7−0.01151 × CD40−0.1905 × FASLG−0.06372 × FLT3−0.01489 × LEF1

### 3.3. The Cox Regression Curve Analysis and Generation of a Nomogram Corresponding to the Necroptosis-Related Risk Model

**Figure 4:**
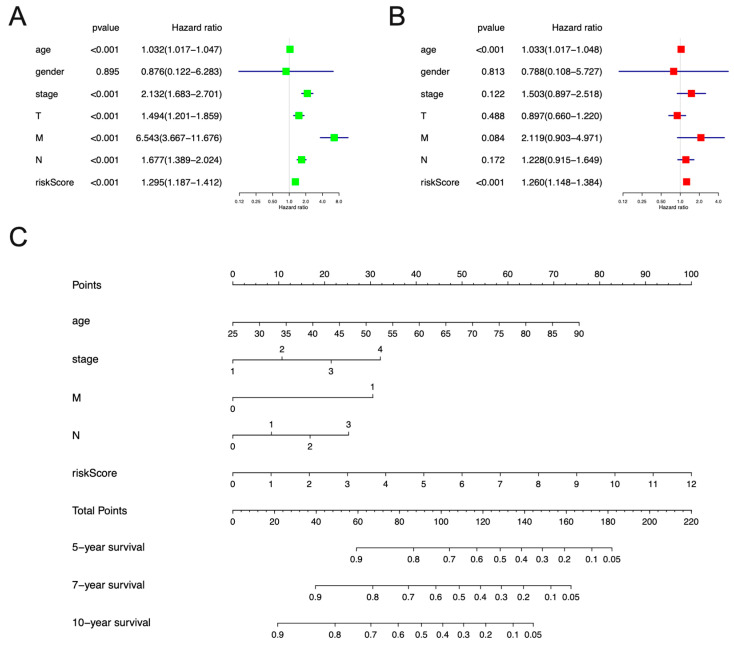
The Cox regression curve analysis and generation of nomogram corresponding to the necroptosis-related risk model. (**A**) The forest plot shows the results of a univariate Cox analysis. (**B**) The forest plot shows the results of a multivariate Cox analysis. (**C**) Based on this necroptosis-related risk model, a nomogram is drawn that predicts 5, 7, and 10-year OS in breast cancer patients.

To examine the role of necroptosis-related risk models and clinicopathological features in breast cancer progression, we performed univariate and multivariate Cox regression analyses. In the univariate Cox regression analysis, age, stage, T, M, N, and riskScore were associated with breast cancer progression (Figure 4A). The multivariate Cox regression analysis showed that age and riskScore were independent risk factors for breast cancer progression (Figure 4B). In addition, based on the necroptosis-related risk model, we created a corresponding nomogram that predicted breast cancer patients’ five-, seven-, and ten-year survival (Figure 4C).

### 3.4. Multi-GSEA Analysis of Necroptosis-Associated Risk Model Genes in BC

**Figure 5:**
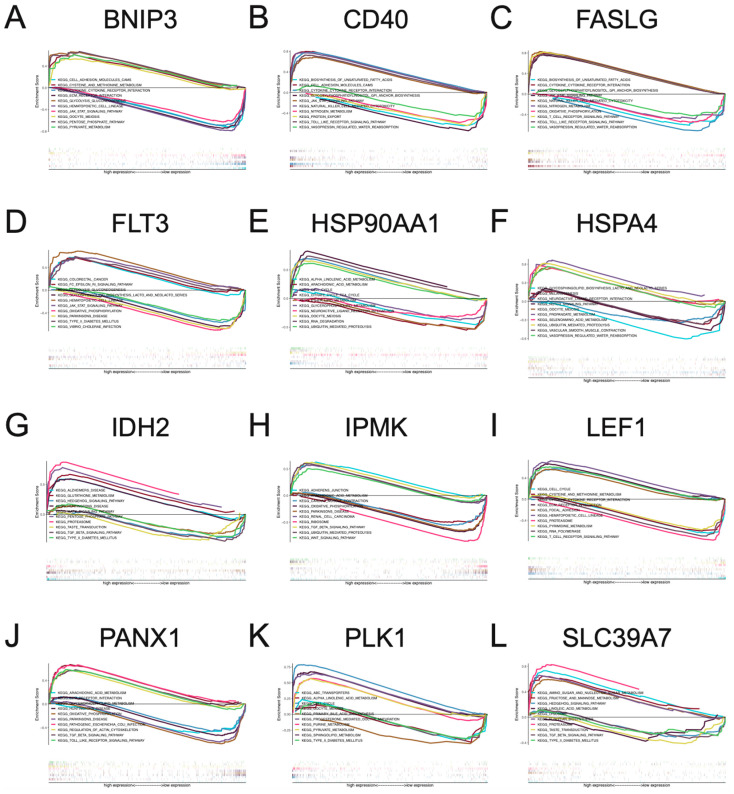
Multi-GSEA analysis of BNIP3 (**A**), CD40 (**B**), FASLG (**C**), FLT3 (**D**), HSP90AA1 (**E**), HSPA4 (**F**), IDH2 (**G**), IPMK (**H**), LEF1 (**I**), PANX1 (**J**), PLK1 (**K**), and SLC39A7 (**L**) in breast cancer.

To understand which biological pathways are linked to necroptosis risk model genes in breast cancer progression, we performed GSEA analysis for these twelve risk model genes. In the GSEA analysis, these twelve risk model genes were significantly correlated with abnormal activation or inhibition of various biological pathways. BNIP3, CD40, FASLG, and FLT3 were associated with abnormal activity in the JAK-STAT SIGNALING PATHWAY. IDH2, IPMK, PANX1, and SLC39A7 were associated with abnormal activity of the TGF BETA SIGNALING PATHWAY. CD40, FASLG, and PANX1 were related to abnormal TOLL LIKE RECEPTOR SIGNALING PATHWAY activity. IDH2 and SLC39A7 were associated with abnormal activity of HEDGEHOG SIGNALING PATHWAY. HSP90AA1, LEF1, and PLK1 are associated with abnormal activity of CELL CYCLE (Figure 5A–L). The biological pathways involved focus on current cancer research to provide meaningful clues for future research.

### 3.5. Pan-Cancer Analysis of Necroptosis-Associated Risk Model Genes

**Figure 6:**
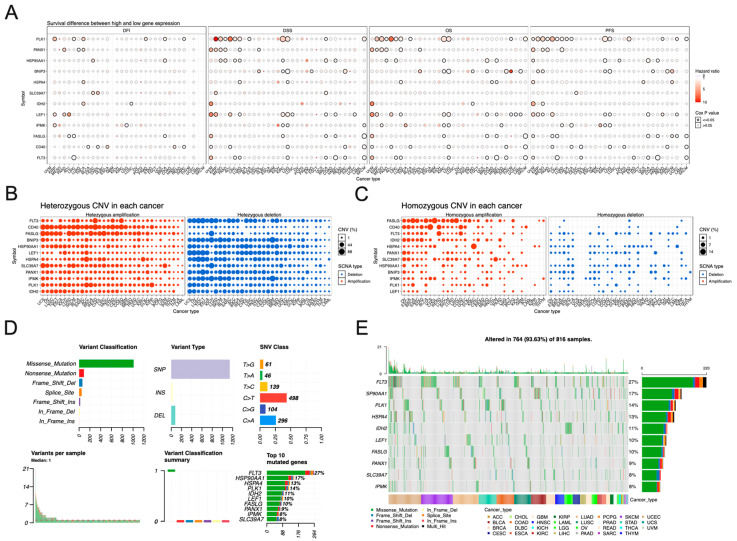
Pan-cancer analysis of necroptosis-associated risk model genes. (**A**) This shows the DFI, DSS, OS, and PFS of these necroptosis risk model genes in pan-cancer. (**B**,**C**) This shows the CNVs of these necroptosis risk model genes in pan-cancer. (**D**,**E**) This shows the SNVs of these necroptosis risk model genes in pan-cancer.

In order to link the clinic closely, we explored the predictive value of these necroptosis risk model genes in various tumor types. The results showed that PLK1, PANX1, BNIP3, LEF1, FASLG, CD40, and FLT3 were significantly correlated with DSS, OS, and PFS in patients with more than five different types of tumors. At the same time, PLK1 and CD40 were significantly associated with DFI in patients with five different types of tumors (Figure 6A). Genetic variations and DNA polymorphisms exist in the human genome [19]. The genome also undergoes micro-duplications and micro-deletions of submicroscopic structures in addition to DNA point mutations [20]. Copy number variation (CNV) exists widely in the human genome, and its range is beyond people’s expectations. The chromosome range covered by CNV accounts for about 12% of the whole human genome [21]. Because of their wide coverage, heritability, relative stability, and high heterogeneity, the genetic mechanism of many diseases is due to CNVs rather than point mutations in individual genes. Its role has received significant attention from researchers [22]. As a result of CNV analysis in pan-cancer targeting of these necroptosis risk model genes, FLT3, CD40, and FASLG genes have high CNVs in various tumor types (Figure 6B,C). Next, we examined the SNV situation of these necroptosis risk model genes in pan-cancer, and the results showed that the top ten SNV variant genes were FLT3, HSP90AA1, PLK1, HSPA4, IDH2, LEF1, FASLG, PANX1, SLC39A7, and IPMK (Figure 6D,E). Among them, FLT3 had up to 27 percent of SNVs in pan-cancer analysis.

### 3.6. The Relationship between Necroptosis-Associated Risk Model Genes and Cancer Signaling Pathways, Drug Sensitivity, and Immune Cell Infiltration

**Figure 7:**
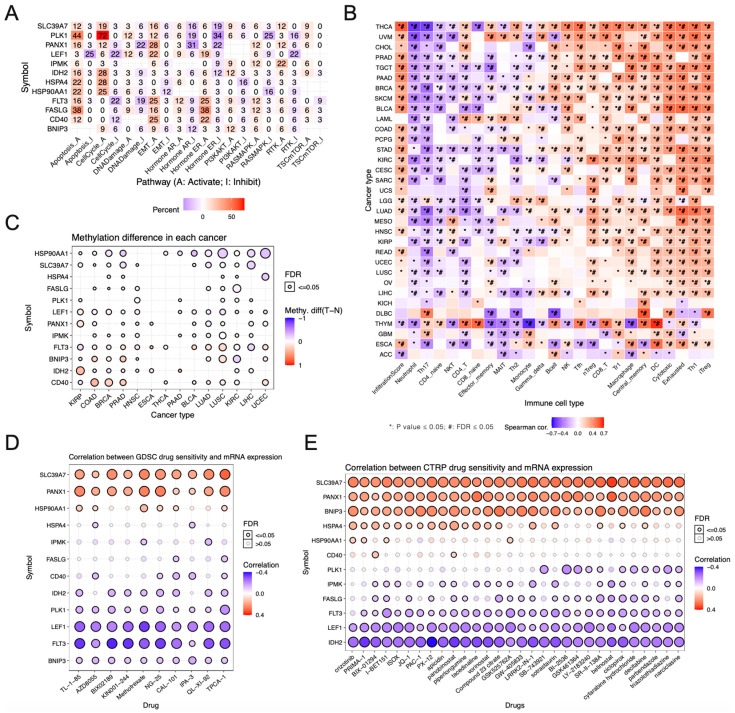
The relationship between necroptosis-associated risk model genes and cancer signaling pathways, drug sensitivity, and immune cell infiltration. (**A**) This heatmap illustrates the correlation between the genes involved in necroptosis risk assessment and the cancer signaling pathways involved. (**B**) A heatmap depicts the relationship between the genes associated with necroptosis risk and the immune cells present in pan-cancers. Notably, red represents a positive correlation and purple represents a negative correlation. * *p* ≤ 0.05 and # FDR ≤ 0.05. (**C**) A comparison of the methylation patterns of these necroptosis risk model genes across several types of tumors is shown here. (**D**,**E**) This table shows the correlation between necroptosis risk model genes and multiple anticancer drug susceptibility in the GDSC and CTRP databases. Red bubbles represent positive correlations, blue bubbles represent negative correlations, and the darker the color, the higher the correlation. The size of the bubbles was positively correlated with FDR significance. Black outline boxes indicate FDR ≤ 0.05.

Afterward, we investigated the relationship between these necroptosis risk model genes and cancer signaling pathways. The results suggested that these necroptosis risk model genes can activate or inhibit multiple important cancer signaling pathways (Figure 7A). PLK1, IDH2, HSPA4, and HSP90AA1 can activate the cell cycle. The cell cycle plays an important role in cell carcinogenesis, and is related to the occurrence, development, recurrence, drug resistance, and prognosis of tumors. It is a potential anti-tumor therapeutic strategy. PANX1, LEF1, FLT3, CD40 can activate EMT. EMT is a concept first proposed by Greenburg in 1982 [23]. EMT means that epithelial cells lose cell polarity under some physiological or pathological factors, tight intercellular junctions, and adhesion junctions, and become cells with the shape and characteristics of mesenchymal cells, thereby gaining the capacity for infiltration and migration [24,25]. Following this, we explored the association between these necroptosis risk model genes and immune cell infiltration in pan-cancers. Combining immune cell infiltration data from the ImmuCellAI database, we created a heatmap reflecting the association between these necroptosis risk model genes and multiple immune cell infiltrations in pan-cancer. The heatmap revealed a significant correlation between these necroptosis risk model genes and most tumor invasion scores (Figure 7B).

We also explored the differences in methylation of these necroptosis risk model genes in various tumor types using TCGA data. These risk model genes showed extensive methylation differences in COAD, BC, PRAD, LUSC, and KIRC (Figure 7C). Depending on the therapeutic target, the response to anticancer drugs varies significantly, as mutations in the cancer genome can affect their efficacy. As a result, we examined the sensitivity of these necroptosis risk model genes to various anticancer drugs using data from the GDSC and CTRP databases. The results showed a significant positive correlation between SLC39A7 and PANX1 and anticancer drug sensitivity. Additionally, there was a significant negative correlation between LEF1 and FLT3 sensitivity to numerous anticancer medications (Figure 7D,E). Our study provides valuable data for future drug target development.

### 3.7. Immunohistochemistry of Necroptosis-Associated Risk Model Genes in BC

**Figure 8:**
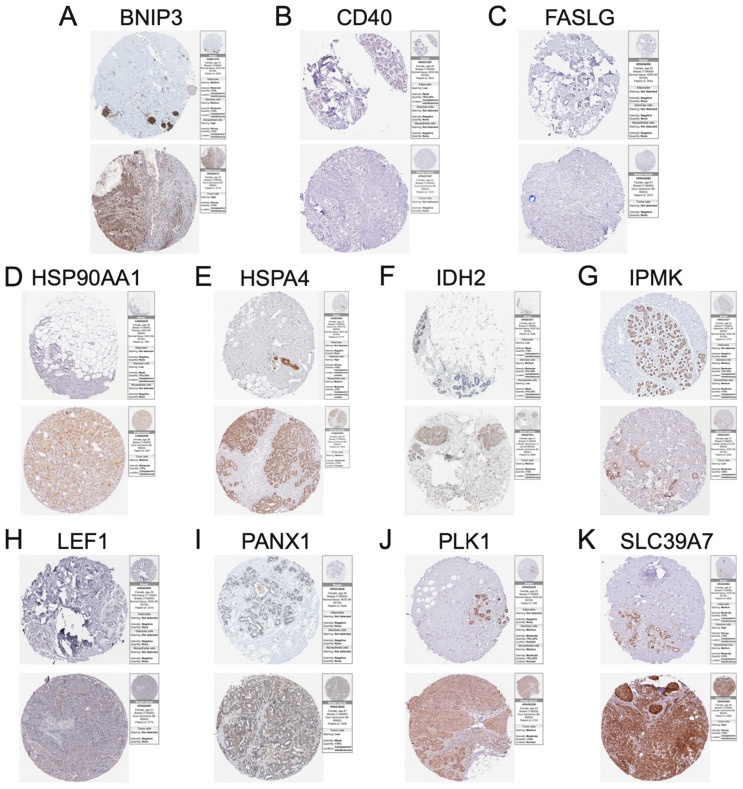
The results of immunohistochemistry showed the protein expression of BNIP3 (**A**), CD40 (**B**), FASLG (**C**), HSP90AA1 (**D**), HSPA4 (**E**), IDH2 (**F**), IPMK (**G**), LEF1 (**H**), PANX1 (**I**), PLK1 (**J**), and SLC39A7 (**K**) in breast cancer tissue and normal breast tissue.

We analyzed immunohistochemical data from the HPA database to examine whether these necroptosis risk model molecules were expressed in breast cancer and normal breast tissues to validate our previous findings. Since FLT3 is not yet included in the HPA database, we present immunohistochemical results for BNIP3, CD40, FASLG, HSP90AA1, HSPA4, IDH2, IPMK, LEF1, PANX1, PLK1, and SLC39A7. Protein expression levels confirmed our previous findings at the mRNA level, verifying our results (Figure 8A–K). We believe this result may provide some inspiration for targeting these necroptosis risk model genes and pave the way for future studies in breast cancer.

## 4. Discussion

BC is one of the most common cancers globally, and the most common malignant tumor in women [26]. Breast cancer is now the most common cancer among women in China, as it is in most other countries, with newly diagnosed breast cancer patients in China accounting for 12.2% of the world’s newly diagnosed breast cancer patients and 9.6% of global breast cancer deaths [27]. It is of great significance for breast cancer treatment to deeply study the mechanism of occurrence and development of breast cancer, and to find new therapeutic targets for breast cancer as well as molecular markers related to prognosis prediction. The body’s growth and development, as well as the cells’ homeostasis, rely heavily on programmed cell death. Necroptosis is mediated by receptor-interacting serine/threonine-protein kinase 3 (RIPK3) and its substrate, mixed lineage kinase domain-like protein (MLKL), of programmed cell death morphologically typical of necrosis. Numerous studies have shown that dysregulation of necroptosis is closely related to human diseases, such as human tumors, inflammatory diseases, autoimmune diseases, and degenerative diseases. This study aimed to conduct an in-depth exploration of necroptosis-related genes in BC and to discover potential new therapeutic targets and biomarkers.

In order to better use bioinformatics methods to complete our research, we refer to a large number of similar studies [28,29,30,31]. This study successfully constructed a novel necroptosis-related risk model that included BNIP3, CD40, FASLG, FLT3, HSP90AA1, HSPA4, IDH2 IPMK, LEF1, PANX1, PLK1, and SLC39A7 genes in BC. BNIP3 shares homology with BCL2 family proteins. BNIP3 has a BH3 domain at its N-terminus and a TM domain at its C-terminus, allowing it to localize to the mitochondrial outer membrane. The apoptosis induced by BNIP3 depends on the TM domain, and the BH3 domain is closely related to its induction of mitophagy. Under normal physiological conditions, BNIP3 is expressed at low levels in the skeletal muscle and the brain, but it is dramatically increased under ischemia and hypoxia [32]. The higher the expression level of BNIP3 in some solid tumors, the higher the degree of tumor necrosis, as well as the gradient changes around the necrotic center, which are closely related to the severity of some ischemia-hypoxic regions in solid tumors. Hypoxia is the main factor for the high expression of BNIP3, and hypoxia-inducible factor 1α (HIF-1α) can directly regulate the expression of BNIP3 [33]. BNIP3 is highly expressed in prostate, lung, endometrial, and breast cancer, and HIF-1α can induce further expression. BNIP3 is closely related to the malignancy of tumors; its expression level is significantly higher in breast ductal carcinoma in situ and invasive breast cancer than in normal breast tissue, and its expression level in lobular invasive lung cancer is substantially higher than that in ductal carcinoma in situ. BNIP3 is highly expressed in liver cancer cells, and activates autophagy to delay cell death [34]. BNIP3 can increase melanoma cells’ migration and invasion ability [35]. Interestingly, BNIP3 is a pro-apoptotic protein that promotes tumor cell apoptosis [36]. Silencing BNIP3 decreases BNIP3-dependent mitophagy and apoptosis [37]. BNIP3 is inactivated by methylation of the CpG island at the transcriptional start site in gastric, pancreatic, and colorectal cancer [38], and methylase inhibitor 5-aza deoxycytidine can restore the deficiency. The expression of BNIP3 under oxygen further induces apoptosis. It can be seen that BNIP3 is closely related to tumor invasion, apoptosis, autophagy, and prognosis. CD40 is abnormally highly expressed in epithelial tumors such as lung, colorectal, breast, ovarian, and melanoma, while its expression is lower in normal tissues [39,40]. The up-regulated expression of CD40 in most epithelial tumors is closely related to its invasion, metastasis, and TNM staging. It may be related to its regulation of tumor cell apoptosis and participation in tumor angiogenesis [41,42].

The HSP90AA1 gene can encode the HSP90α protein. HSP90α is a chaperone protein that plays a vital role in the occurrence and development of cancer [43], and has also been confirmed to have a variety of biological functions in cancer [44,45]. Some experiments have reported that the expression level of HSP90α in the blood of breast cancer patients was increased [46]. HSP90AA1 can induce tumor angiogenesis by regulating the expression of vascular endothelial growth factor (VEGF) and promoting tumor proliferation and metastasis [47,48]. PANX1 is a subtype of the Pannexin gene, a new member of the gap junction family which was discovered in recent years, and also includes two other subtypes of PANX2 and PANX3 [49]. The six subunits of PANX1 form a hexamer. Its primary function is to form a macroporous single-membrane channel, which can be activated by stimuli such as voltage, high intracellular calcium, and high extracellular potassium. The PANX1 channel plays a regulatory role in cellular communication and signal transduction [50,51]. Studies have shown that PANX1 is involved in the occurrence and development of cell proliferation, cell differentiation, inflammation, cerebral ischemia, epilepsy, and tumors [52,53,54]. PLK1 is highly expressed in various tumors, including lung, head and neck, esophageal, gastric, melanoma, breast, ovarian, endometrial, colorectal, glioma, renal cell carcinoma, and thyroid cancer [55,56]. Smith et al. transfected exogenous PLK1 into normal fibroblasts, leading to malignant transformation of cells and inducing tumorigenesis in nude mice, suggesting that PLK1 can directly affect the malignant transformation of cells. PLK1 expression was also found to be high in early stages of liver and pancreatic cancers [57]. In addition, PLK1 can also inhibit the activity of p53 by phosphorylating G2/S-expressed protein 1 and Topors (a ubiquitinated suMO E3 ligase), helping DNA-damaged cells to escape checkpoint blockade [58]. On the one hand, overexpression of PLK1 can directly promote cell proliferation. On the other hand, it can also increase chromosomal instability by assisting DNA-damaged cells in escaping the blockade of checkpoints, and ultimately leading to tumorigenesis.

The role of necroptosis-related genes in breast cancer, such as FASLG, FLT3, HSPA4, IDH2, IPMK, LEF1, and SLC39A7, has not been extensively studied, and these necroptosis-related genes have been confirmed to regulate the biological processes of necroptosis in various types of tumors. In addition, many studies on these necroptosis-related genes only involve the cell culture level, so it is more important to use animal models to define whether and how these necroptosis-related genes regulate the progression of necroptosis in vivo. Research on necroptosis improves breast cancer’s poor prognosis, and chemotherapy resistance is gradually reported. Still, more in-depth clinical studies are lacking to confirm its feasibility and safety. In addition, this study still has certain limitations. Although the newly proposed necroptosis-associated risk model has apparent clinical significance in breast cancer, its underlying mechanism remains unclear. Therefore, we plan to further explore the role of necroptosis-associated risk models in single-center or multi-center clinical samples. It is believed that with the continuous deepening of necroptosis research, more therapeutic targets will be provided for the treatment of breast cancer patients in the future, and the poor prognosis of breast cancer patients will be significantly improved.

## 5. Conclusions

This study used necroptosis-related genes to construct a novel risk signature in breast cancer that could predict OS time in breast cancer patients. Based on this necroptosis-related risk model, we can easily divide breast cancer patients into high-risk and low-risk groups in clinical practice. For breast cancer patients in the high-risk group, we can choose to intensify treatment, increase the frequency of follow-up appropriately, and pay close attention to the progress of the breast cancer. We believe this will be very helpful in prolonging the survival time of breast cancer patients in the high-risk group. Furthermore, we provide new insight into the relationship between necroptosis-related genes and breast cancer. We should examine the validity of our model in future studies so that, one day, it can be applied to clinical diagnosis and treatment.

## Data Availability

The data used to support the findings of this study are available from the corresponding author upon request.

## Supplementary Materials

The following supporting information can be downloaded at: https://www.mdpi.com/article/10.3390/jpm13010044/s1. Table S1: The table of necroptosis-related genes refers to the GSEA database and previous literature reports.

## Abbreviations

BC: Breast Cancer; TCGA: The Cancer Genome Atlas; GSCA: Gene Set Cancer Analysis; THPA: The Human Protein Atlas; ImmuCellAI: Immune Cell Abundance Identifier; GDSC: Genomics of Drug Sensitivity in Cancer; LASSO: Least Absolute Shrinkage and Selection Operator; GSEA: Gene Set Enrichment Analysis; HSP90AA1: Heat Shock Protein 90 Alpha Family Class A Member 1; PANX1: Pannexin 1; BNIP3: BCL2 Interacting Protein 3; FLT3: Fms Related Receptor Tyrosine Kinase 3; SLC39A7: Solute Carrier Family 39 Member 7; TNFRSF1B: TNF Receptor Superfamily Member 1B; CD40: CD40 Molecule; IPMK: Inositol Polyphosphate Multikinase; PLK1: Polo Like Kinase 1; HSPA4: Heat Shock Protein Family A (Hsp70) Member 4; FASLG: Fas Ligand; LEF1: Lymphoid Enhancer Binding Factor 1; MLKL: Mixed Lineage Kinase Domain-Like Protein; RIPK3: Receptor Interacting Serine/Threonine Kinase 3; YAP: Yes1 Associated Transcriptional Regulator; TAZ: Tafazzin, Phospholipid-Lysophospholipid Transacylase.
